# Andrographolide: A Novel Antimalarial Diterpene Lactone Compound from *Andrographis paniculata* and Its Interaction with Curcumin and Artesunate

**DOI:** 10.1155/2011/579518

**Published:** 2011-06-07

**Authors:** Kirti Mishra, Aditya P. Dash, Nrisingha Dey

**Affiliations:** ^1^NVBDCP (Orissa State), Bhubaneswar 751001, India; ^2^Regional Advisor, Vector-Borne Disease Control, World Health Organization, Regional Office for South East Asia, World Health House, Indraprastha Estate, Mahatma Gandhi Marg, New Delhi 110 002, India; ^3^Institute of Life Sciences, Nalco Square, Chandrasekharpur, Bhubaneswar 751 023, Orissa, India

## Abstract

Andrographolide (AND), the diterpene lactone compound, was purified by HPLC from the methanolic fraction of the plant *Andrographis paniculata*. The compound was found to have potent antiplasmodial activity when tested in isolation and in combination with curcumin and artesunate against the erythrocytic stages of *Plasmodium falciparum in vitro* and *Plasmodium berghei* ANKA *in vivo*. IC_50_s for artesunate (AS), andrographolide (AND), and curcumin (CUR) were found to be 0.05, 9.1 and 17.4 **μ**M, respectively. The compound (AND) was found synergistic with curcumin (CUR) and addictively interactive with artesunate (AS). *In vivo*, andrographolide-curcumin exhibited better antimalarial activity, not only by reducing parasitemia (29%), compared to the control (81%), but also by extending the life span by 2-3 folds. Being nontoxic to the *in vivo* system this agent can be used as template molecule for designing new derivatives with improved antimalarial properties.

## 1. Introduction

The discoveries leading to information on novel drugs and drug combinations have become a priority to manage the increasing burden of malaria, caused by drug resistant parasites. Artemisinin derivative-based drug combinations that have independent mode of action are seen as a way of enhancing efficacy and simultaneously ensuring protection against resistance [1, 2] among the parasites. The anti-malarial properties of a tropical plant *Andrographis paniculata *has been studied in details recently by Mishra et al. [3], and also, there are several reports that demonstrate the antimalarial properties of the plant *A. paniculata *[4–6], but there is hardly any report that describe the active compound responsible for the anti-malarial property of this plant. Andrographolide, the diterpenic lactone compound, is one of the major phytoconstituents of the plant *A. paniculata* and has been reported to have diverse pharmacological potential including antiviral [7], anti-inflammatory [8–11], and anticancer properties [12]. The present study for the first time establishes andrographolide as the major bioactive anti-malarial constituent of the plant *A. paniculata* using both *in vitro* and *in vivo* approaches. The encouraging *in vitro* antiplasmodial activities of andrographolide prompted us to evaluate the interaction of andrographolide (AND) with other established anti-malarial compounds such as curcumin (CUR) and artesunate (AS), the hemisuccinate derivative of artemisinin *in vitro* on *Plasmodium falciparum,* and *in vivo* on* Plasmodium berghei *ANKA strains. This adds new information on combination of potent anti-malarial compounds for novel combinatorial drug therapy against drug-resistant malaria.

## 2. Methods

### 2.1. Compounds

Pure grade andrographolide, (Sigma-Aldrich, USA; Cat no. 365645), artesunate (Sigma, USA; Cat no. 3731-100MG) and curcumin (98% Curcuminoid content, Sigma, USA; Cat no. 7727) were purchased commercially.

### 2.2. Parasite Strain and In Vitro Culture

 Chloroquine-resistant strain of *Plasmodium falciparum* (MRC-pf-303) was obtained from the Malaria Parasite bank, maintained by the National Institute of Malaria Research, New Delhi. Parasites were cultured in human O^+^ washed erythrocytes using standard protocols [13]. The parasite cultures were synchronized by treatment with 5% D-Sorbitol [14]. A tightly synchronized culture was obtained by two sorbitol treatments that were carried out 12 h apart. The residual schizonts were eliminated by resynchronization, 48 h after the initial synchronization.

### 2.3. Preparation of Bark Extract

The aerial parts of the plant *A. paniculata* were crushed in liquid N_2_ to fine powder. Crude extracts were prepared as per the method reported earlier [3]. Usually, a 0.4% yield of crude extract was obtained invariably from each extraction. 

### 2.4. Isolation of Andrographolide from the Bark Extract

**Figure 1:**
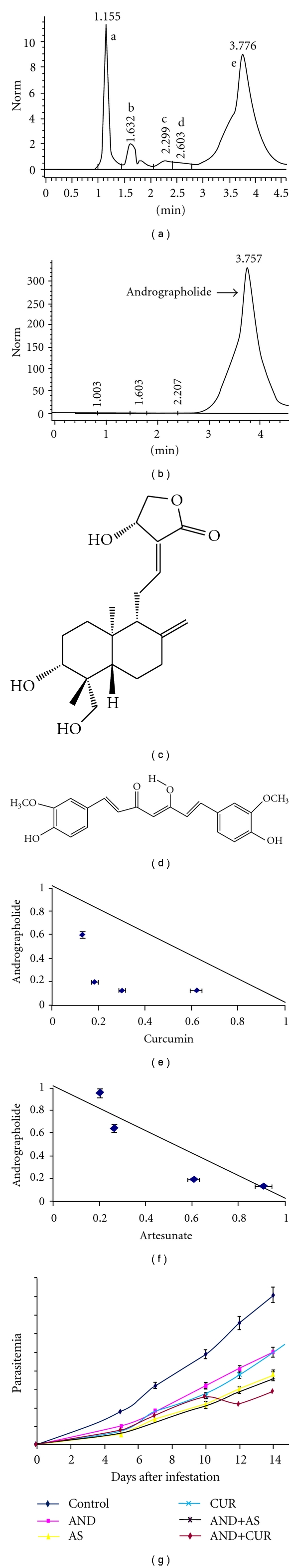
(a) Chromatogram showing purification of andrographolide (AND, retention time, 3.776) from crude bark extracts of *Andrographis paniculata. *(b) Chromatogram showing the peaks for andrographolide (retention time, 3.757) in standard andrographolide. (c) Structure of andrographolide. (d) Structure of curcumin, (e) and (f) Representative isobolograms of the interactions between andrographolide with curcumin (e) and andrographolide with artesunate (f) against chloroquine-resistant strains (MRC-pf-303) of *P. falciparum*. The values in *X*- and *Y*-axes of the isobolograms represent the FIC_50_ values of andrographolide-curcumin and andrographolide-artesunate, respectively, at different concentration ratios (1 : 5, 1 : 2, 2 : 1, and 5 : 1). (g) *In vivo* efficacy of andrographolide (AND), curcumin (CUR) and artesunate (AS), tested in isolation and combination in *Plasmodium berghei* ANKA-infected mice.

Filter-sterilized crude herbal extracts of *A. paniculata* were separated by HPLC, using C18 column (Agilent Technologies, 1200 series) in UV range (254 nm) and at 25°C temperatures. Fixed ratio of methanol : H_2_O (1 : 1) was used as the mobile phase to sieve the phytoconstituents present in the extracts at a flow rate of 1 mL/min as described by Mishra et al. [15]. Different phytoconstituents that were eluted under different peaks a, b, c, d, and e (Figure 1(a)), were collected and pooled. The solvent part (methanol) was evaporated to dryness at 37°C by the help of a Speed Vac (Model 7811001, Labconco, USA), and the compounds were assayed individually on *Plasmodium falciparum* (MRC-pf-303) for their *in vitro* antiplasmodial activity.

### 2.5. In Vitro Antiplasmodial Activity and Drug-Interaction Tests

Dose-response assays and *in vitro* drug-interaction studies were carried out to obtain the 50% inhibitory concentrations (IC_50_) and 50% fraction inhibitory concentrations (FIC_50_) of these bioactive compounds, AND, CUR, and AS, respectively, as described earlier [3, 15]. Corresponding isobolograms (Figures 1(d) and 1(e)) were constructed using the method reported earlier [3, 16].

### 2.6. In Vivo Toxicity Study

*In vivo* toxicity study was carried out using Balb/C mice (20 ± 3 g, *n* = 4). Animals were injected intraperitoneally with the test compounds affording doses of 50 mg/kg of body weight with one group being given DMSO (DMSO control) in the same concentration along with serum-free medium, while the other group of control was continued without any solvent treatment. Mice were observed for 96 h to record postinjection discomfort, if any. At the end of 4th day, the gross body weight was recorded. Animals were bled through heart puncture, and the blood, and serum were subjected for hematological and physiological analyses.

### 2.7. In Vivo Assay of Test Compounds on *P. berghei* ANKA Strain

*In vivo* anti-malarial activity was carried out using 5 groups of mice (Balb/C, 28 g ± 4 g, *n* = 4). Mice were injected intraperitoneally with *P. berghei* ANKA-infected mouse blood (approximately 40% parasitemia) on day 1, at a density of 1 × 10^7^ parasites (approximately) of the strain. The test compounds (AND, AS, CUR, AND+AS, and AND+CUR) were dissolved in DMSO and serum-free media to make the desired concentrations and were injected (15 mg/kg of body weight) intraperitoneally to individual groups of animals starting from 24 h prior to the parasite challenge (day 0). The control mice were maintained simultaneously without any treatment. Injections were continued daily till the death of the animals in the control group (13–15 days). Parasitemia levels (parasitized erythrocytes/total erythrocytes) were determined with thin blood smears on every alternate day from day 5 onwards of posttreatment (Figure 1(f)). The animal experiments complied with all relevant guidelines and institutional policies on animal ethics and were conducted under environmentally controlled condition, as reported earlier [3].

## 3. Results

### 3.1. Purification and Identification of Andrographolide

The phytoconstituents eluted under different peaks were assayed individually *in vitro* on *Plasmodium falciparum*. The particular phytoconstituent eluted under peak “e” (retention time 3.78) showed potent anti-malarial activity compared to the compounds eluted under peaks a, b, c, and d (Figure 1(a)). The compound of peak “e” was later identified as andrographolide by comparing the retention time of pure andrographolide (Sigma, Cat no. 365645) which was run as positive control (Figure 1(b)) and finally by using standard methods of compound identification [15, 17, 18]. Approximately, 50 cycles of HPLC purification were carried out to obtain a sufficient amount of test compound, andrographolide (Figure 1(c)) for their *in vitro* anti-plasmodial assays.

### 3.2. IC_50_ Values of AS, AND, and CUR

IC_50_s for artesunate (AS), andrographolide (AND), and curcumin (CUR) were found to be 0.05, 9.1, and 17.4 *μ*M, respectively. It was noted that the compounds are particularly inhibiting the parasites at their ring stage.

### 3.3. In Vitro Drug Interaction

The fraction IC_50_s (FIC_50_) of the combinations made out of the three compounds was found much below the level of their individual IC_50_s, as expected. The sums of the FIC values (*∑* FIC) are an important parameter that detects and differentiates the classification of the drug interaction. When the sum of putative drug fractions is 1, the combination is said to be additive; when the sum is <1, the combination is synergistic; and when the sum is >1, the combination is antagonistic [19]. Curcumin (Figure 1(d)) was found to be a better combination partner with andrographolide as compared to artesunate with sum FIC_50_s in the former combination (AND+CUR) remaining within 1 (0.73, 0.38, 0.43, and 0.75) in all 4 ratios (1 : 5, 1 : 2, 2 : 1, and 5 : 1), respectively. This combination (AND+CUR) at lower ratios (1 : 2 and 2 : 1) yielded synergism, while higher ratios (1 : 5 and 5 : 1) exhibited additiveness as shown by the isobologram (Figure 1(e)). Alternatively, AND+AS was found effective only at lower ratios (1 : 2 and 2 : 1) with proven additivity, (sum FIC_50_s being 0.9 and 0.8), respectively. This combination (AND+AS) exhibited indifference with sum FIC_50_s exceeding 1 (1.15 and 1.04) at higher ratios (1 : 5 and 5 : 1), respectively (Figure 1(f)).

### 3.4. In Vivo Antimalarial Activity of Test Compounds without Any Toxicity

The results indicated that andrographolide performed better anti-malarial activity when combined with curcumin than artesunate. All the mice died after attaining the parasitemia level 80% ± 4%. Control group, which died between 13 to 15 days (maximum deaths being on 14th day), were found to harbor 81% ± 2% parasitemia compared to the experimental groups treated with AND, AS, CUR, AND+AS, and AND+CUR, carrying load of parasitemia, 46%, 33%, 46%, 36%, and 29%, respectively (Figure 1(f)). All the treatment groups were kept under observation, and the % parasitemia were monitored till the 30th day (data not shown). 

Because of the interesting *in vitro* anti-malarial properties, *in vivo* toxicity tests were carried out with mice by intraperitoneal routes during four consecutive days. No mortality or any other abnormality was observed during the treatment period. Treated mice were clinically healthy, and no toxic effects and weight loss were observed with the treatment groups even after 96 hrs. The levels of SGOT (serum glutamic oxaloacetic transaminase) and SGPT (serum glutamic pyruvate transaminase) and the blood parameters of the experimental and control mice were found within the normal range without any significant variation (SGOT, 60.3 ± 3.1; SGPT, 74 ± 4.4 U/mL; urea, 19.6 ±2.1 mg/dL; glucose, 90 ± 3.2 mg/dL; creatinine, 0.77 ± 0.16 mg/dL).

## 4. Discussion

Andrographolide is a labdane diterpene reported to have potent anti- inflammatory, anticancer and anti-viral activity [9]. As such, the use of antibacterials and antivirals in malaria prophylaxis is also well documented [20]. Identification of anti-plasmodial activity of this compound in view of its efficacious *in vitro* and *in vivo* performance opens up several opportunities for further exploration of this compound as a new anti-malarial. 

During the erythrocytic life cycle, the period of activity of andrographolide was found evidently on the ring stage of the parasite. The point of action of this compound in the parasite life cycle corresponds with the protein and nucleic acid synthesis. Hence, they could be the “transcription blockers”; more studies focusing on this particular aspect are in progress.

The pharmacological targets of this compound and its mechanism of action on *P. falciparum* are not known. However, the clue centers on the phenomenon of regulation of a transcription factor. As per Hidalgo et al. [9], the anti-inflammatory action of andrographolide includes inhibition of the nuclear transcription factor-(kappa) B, making it a therapeutic target for the treatment of cancer and autoimmune diseases. The role of NF-kappaB (NF-*κ*B) is also important in malaria as mentioned in a recent study [21]; *P. falciparum* infected erythrocytes have shown to induce NF-(kappa) B-regulated inflammatory pathways in human cerebral endothelium. The deciphering of anti-malarial activity in andrographolide against the blood stage of the plasmodial life cycle lays foundation to reevaluate its possible role in the regulation of this important transcription factor for the effective control of malaria. 

There are several reports on various drug combinations showing their *in vitro* and *in vivo* interactions on malaria parasites [16, 22, 23]. The synergy between andrographolide and curcumin is interesting in the fight against the emergence of drug resistance among parasites. Both curcumin and andrographolides being from the plant sources are already documented for their antioxidant [24] and anti-inflammatory properties [25], respectively. This information also generates scope to explore the possibility of modifying the molecular structures of both the plant-derived compounds such as andrographolide and curcumin with the objective of increasing their specific activity against plasmodial species. The semisynthetic modification of artemisinin to artesunate, artemether, and DHA has already yielded compounds with significantly higher activity. The other finding that deciphers the additiveness of andrographolide with artesunate, the most potent artemisinin derivative makes it important in the context that it offers opportunities to further standardize new ACT- (artemisinin-based combination therapies-) based formulae as possible antimalarial combination. 

Andrographolide exhibited high level of activity in murine model when administered either with curcumin or artesunate by the intraperitoneal route, without any toxicity. The substantial decrease in level of parasitemia in response to test drug combinations compared to the control and extended periods of life, observed with the mice is the main finding of the present study. This may be attributed to the observed *in vitro* synergism or addictiveness between the putative drug compounds in different combination ratios. However, studies are in progress for effective dose determination for complete clearance of parasitemia from infected mice using AND+CUR and AND+AS combinations.
